# miR-512-5p Suppresses Tumor Growth by Targeting hTERT in Telomerase Positive Head and Neck Squamous Cell Carcinoma *In Vitro* and *In Vivo*

**DOI:** 10.1371/journal.pone.0135265

**Published:** 2015-08-10

**Authors:** Jun Li, Han Lei, Yong Xu, Ze-zhang Tao

**Affiliations:** 1 Department of Otolaryngology-Head and Neck Surgery, Zhongnan Hospital of Wuhan University, Wuhan, 430071, China; 2 Department of Otolaryngology-Head and Neck Surgery, Renmin Hospital of Wuhan University, Wuhan, 430060, China; 3 Hubei key laboratory of Tumor Biological Behaviors, Zhongnan Hospital of Wuhan University, Wuhan, 430071, China; University of Cincinnati, College of Medicine, UNITED STATES

## Abstract

Telomerase activation has very important implications for head and neck squamous cell carcinoma (HNSCC), but the regulatory mechanisms of telomerase in HNSCC remain unclear. In our present study, we found that miR-512-5P was markedly downregulated in telomerase-positive HNSCC cell lines. Both in vitro and in vivo assays revealed that miR-512-5P mimic attenuated HNSCC cell proliferation, and tumor growth in nude mice, which exerts its tumor suppressor function through elevated apoptosis, inhibition of the telomerase activity, decrease of telomere-binding proteins and shortening of telomere length by human telomerase reverse transcriptase (hTERT) downregulation. Furthermore, the dual-luciferase reporter gene assay results demonstrated that hTERT was a direct target of miR-512-5P. We conclude that the frequently miR-512-5P overexpression can regulate hTERT and function as a tumor suppressor in HNSCC. Therefore, miR-512-5P may serve as a potential therapeutic agent for miR-based HNSCC therapy.

## Introduction

Head and neck squamous cell carcinoma (HNSCC) describes a broad range of various carcinomas including orbits, nasopharynx, oropharynx, oral cavity, hypopharynx, and larynx[1]. Worldwide, the incidence of HNSCC exceeds half a million annually, making it the fifth most common cancer diagnosed every year, which is a definitely important contributor to cancer related deaths[1]. In the past 20 years, despite recent marked progresses in diagnose and treatment of HNSCC, such as surgery, radiotherapy or chemotherapy, almost 30–50% of patients will undergo regional recurrence and distant metastases after the comprehensive treatments [2]. Therefore, searching for basic science research and development of novel therapeutic agents for HNSCC is significant and exciting.

Telomerase, expressed in more than 90% of human malignancies, is a multi-component ribonucleoprotein located at the nucleus, synthesize the repetitive nucleotide sequence forming the telomeres at the end of chromosomes[3]. In the telomerase functions, telomerase-dependent replicative immortality is one of the hallmarks of cancer progression in most human tumors, other telomerase function include the cell proliferation stimulation, protection against DNA damage and apoptosis, modulation of specific gene expression and tumor progression[4, 5]. These features of telomerase indicates that measurement of telomerase may be useful both in the diagnosis of malignancy or as a prognostic indicator in cancer treatment[6]. For HNSCC patients, telomerase activation, as significant clinical usefulness marker, may be helpful in predicting the clinical outcome and thus in identifying the need of adjuvant treatment in the HNSCC patients [7, 8]. However, the mechanism of the initial telomerase activation and regulation in HNSCC remain unclear.

MicroRNAs(miRs) are a class of short endogenous noncoding RNA molecules that participate in the post-transcriptional regulation of gene expression by base pairing with the 3’-UTR of target messenger RNAs (mRNAs), which results in degradation or repression of the target mRNAs and inhibits gene expression[9]. Emerging research has revealed miRs regulate fundamental pathophysiological processes of HNSCC, such as differentiation, proliferation, apoptosis and metabolism[9]. Recent reviews have shown that aberrant expression of miRs has proved to be a clinically useful diagnostic or prognostic molecule biomarker[10]. Non-coding RNAs, such as miR-138-5p, have also been implicated in the regulation of telomerase, which was shown to regulate telomerase activity in thyroid carcinoma cells [11], similar effects can also be seen through miR-181a regulated tumor-specific anti-cancer effects in liver cancer[12]. Therefore, we hypothesized that miRs may play an important role in regulation of telomerase and mediate oncogenesis in telomerase-positive HNSCC.

The aim of the present study is to identify a distinct miR expression signature for HNSCC with various telomerase status in order to unveil individual miRs that may be involved in the hTERT regulation and cell growth and survival of telomerase-positive HNSCC.

## Materials and Methods

### Ethics statement

All animal procedures were approved by the Institutional Animal Care and Use Committee (IACUC) of the Wuhan University School of Medicine (Wuhan, China) in accordance with the regulations of the National Institute of Health and all details of animal welfare were in accordance with the recommendations of the Weather all report. The animals were housed in an air-conditioned room with an ambient temperature of 16–26°C, a relative humidity of 40–70% and a 12-hour light-dark cycle at the Animal Bio-Safety Level-III (ABSL-III) laboratory of the Wuhan University School of Medicine which were monitored by a computer-based recording system. The animals were individually housed in stainless steel wire-bottomed cages and allowed access to standard chow diet and water. Animal health was monitored daily by the animal care staff and veterinary personnel. Procedures were carried out carefully to minimizing suffering and stopped to cease pain if necessary. All efforts were made to minimize suffering.

### Cell culture and transfection of miR-512-5P mimic

Cells from Hep-2 (human laryngeal carcinoma cell line), CNE (human nasopharyngeal carcinoma cell line), U-2 OS (human osteosarcoma cell line) and BEAS-2B (human lung epithelial cell line) were obtained from the Chinese Academy of Science Cell Bank (Shanghai, China), which were maintained in RPMI 1640 supplemented with 10% fetal bovine serum (Hyclone, USA) and 100 units/mL penicillin/streptomycin (Sigma-Aldrich, USA) at 37°C in 5% CO_2_. MiR-512-5p mimic and miR scramble were synthesized and purified by Guangzhou Ribobio. Transfection was performed with lipofectamine 2000 Reagent (Invitrogen, USA), following the manufacture’s protocol. Briefly, cells were seeded into 6 well plates one day prior to transfection. When the cells reached 50–70%, miRs were transfected into the cells at a flnal concentrations of 50 nM.

### MicroRNA array

Total RNA was harvested using Trizol Reagent (Invitrogen, USA) and RNeasy mini kit (QIAGEN, Germany) according to manufacturer’s instructions. After having passed RNA measurement on the Nanodrop instrument, the samples were labeled using the miRCURY Hy3/Hy5 Power labeling kit (Exiqon, USA) and hybridized on the miRCURY LNA Array (v.11.0). Scanning was performed with the Axon GenePix 4000B microarray scanner. GenePix pro V6.0 was used to read the raw intensity of the image.

### MiRNA qRT-PCR analyses

Real-time PCR analysis for miR-512-5p was performed using the TaqMan miR Kit (Applied Biosystems, USA). U6 RNA was used as an endogenous control for miRNA detection. The miR-512-5p primer sequences were as follows: 5’-CCTGGCCAATATGGTGAAACC-3’ (forward) and 5’-TGCCATGGAGCGATCATCAG-3’(reverse). The relative expression was calculated using the comparative threshold cycle(Ct) method. Polymerase chain reaction analysis was performed using gene-specific primers.

### Cell proliferation assay

For cell proliferation assay, the cells of each group were reseeded in 96-well plates 24 h post-transfection and incubated in 150 mL culture medium overnight. Twenty-four hours later, cells were stained with 3-(4, 5-dimethylthiazol-2-yl)-2, 5-diphenyltetrazolium bromide (MTT) (Sigma, USA), and incubated at 37°C for 6 h. After removal of the supernatant, 150ml dimethylsulphoxide (Sigma, USA) was added and thoroughly mixed for 15 min. MTT assay was performed to detect cell viability at 1, 2, 3, 4, and 5 days and the absorbance was measured at 490 nm with a spectrophotometric plate reader.

### Apoptosis assay

All cells were collected 48 hours post-transfection for apoptosis analysis and then stained with Annexin V-FITC (5 ml) and propidium iodide (5 ml) using the Annexin V-FITC Apoptosis Detection Kit (Beyotime, China). The percentage of apoptotic cells was counted using a Flow cytometry (Beckman, USA). All analyses were performed in triplicate.

### Relative telomere length measurement

Relative telomere length was detected by the novel approach raised by Cawthon previously[13]. Genomic DNA of the cancer cells was extracted using TIANamp genomic DNA kit (TIANGEN, China). The mean telomere sequence to a reference gene is expressed as T/S ratio which calculated to evaluate the relative telomere length. Duplicate PCR reactions were executed using the TaqMan miR Kit (Applied Biosystems, USA) according to the manufacturer’s instructions. The specific primer sequences of telomere and single copy gene used for the examination were given as follows: Tel A: 5’-GTTTTTGAGGGTGAGGGTGAGGGTGAGGGTGAGGT-3’; TelB: 5’-TCCCGACTATCCCTATCCCTATCCCTATCCCTATCCCTA-3’;36B4A:5’-CAGCAAGTGGGAAGGTGTAATCC-3’,36B4B:5’-CCCATTCTATCATCAACGGGTACAA-3’. The cycling conditions consisted of a pre incubation for 5 sec at 95°C, followed by 35 cycles of 15 sec at 95°C and 2 min at 54°C. PCR reactions were worked up by aliquoting 15 μl of master mix into each reaction using the MX3000P (Stratagene, USA) Detection System and the result was proposed using the MX3000P analysis software. All experimental samples were assayed in triplicate.

### Telomerase activity measurement

The telomerase activity was determined using the PCR-TRAP ELISA kit (Roche, USA) according to the manufacturer’s description. Sample absorbance was measured with Model 550 Microplate Reader (Bio-Rad, USA) at the wavelength of 450/690 nm within 30 min after addition of the stop reagent.

### Western hybridization

Proteins extracted from the cells were analyzed by SDS-polyacrylamide gelelectrophoresis (SDS-PAGE) and transferred to nitrocellulous membrane. The hTERT or TRF2 protein was detected with an anti-hTERT or anti-TRF2 polyclonal antibody (Santa Cruz, USA) at a 1:800 dilution. Polyclonal anti-GAPDH antibody (Santa Cruz, USA) was an internal loading control.

### The prediction of potential targets of miR-512-5p and luciferase reporter assay

MiR target site prediction for hTERT was performed using Targetscan Release 6.2., PicTar5 and miRanda 3.0 programs. The hTERT gene was predicted by all programs and for further validation. The hTERT and mut-hTERT sequence were individually sub-cloned downstream to the luciferase coding sequence in the the 3’UTR region of pMIR-Report construct (Life Technologies, China). About 1×10^4^ 293T cells were seeded into each well of 24-well plate and co-transfected with wide type or mutant reporter constructs together with miR-scramble or miR-512-5p using the lipofectamine 2000 (Invitrogen, USA). After 48h incubation, luciferase activities were measured using Dual-Luciferase Reporter Assay System (Promega, USA) following the manufacturer’s instructions. All the data was normalized by dividing firefly luciferase activity with that of Renilla luciferase.

### Tumor xenografts in nude mice

Female BALB/c-nude mice (Wuhan University Laboratory Animal, China) aged 8 weeks were used for tumor xenografts. The nude mice were then randomized and divided into two groups, CNE-scramble group and CNE-miR-512-5p mimic group, respectively. CNE cells treated with scramble or miR-512-5p mimic (150 nM for 24 h) were injected into the nude mice. Tumor width (W) and length (L) were measured and mice with anesthetization by isoflurane were sacrificed 5 weeks post-injection and tumors from the two groups were extracted and weighed. Tumor size were measured weekly by the modified ellipsoid formula: (π/6) ×LW2.

### Immunohistochemistry (IHC)

Tissue samples were deparaffinized in xylene and rehydrated in graded alcohols and distilled water after that slides were processed for antigen retrieval by a standard microwave heating technique. Specimens were incubated with anti-hTERT antibodies (Santa Cruz, USA) with 1:500 dilution. The immunoreactions were visualized with diaminobenzidine and counterstained with hematoxylin. The slides were washed and in tap water and dehydrated in alcohol. The stained sections were examined and photographed on a Optiphot II microscope with a camera (Nikon, Japan).

### Statistical analysis

Each experiment was conducted in triplicate and all experiments were repeated three times and al data was analyzed with SPSS 17.0 software. The results are presented as the mean ± standard deviation. The Student’s t-test was used to evaluate the significant difference of two groups of data in all the pertinent, and One Way ANOVA was used among three groups of date. A two sided p < 0.05 was considered significant.

## Results

### MiR-512-5P is markedly down-regulated in telomerase positive HNSCC cell lines

**Fig 1:**
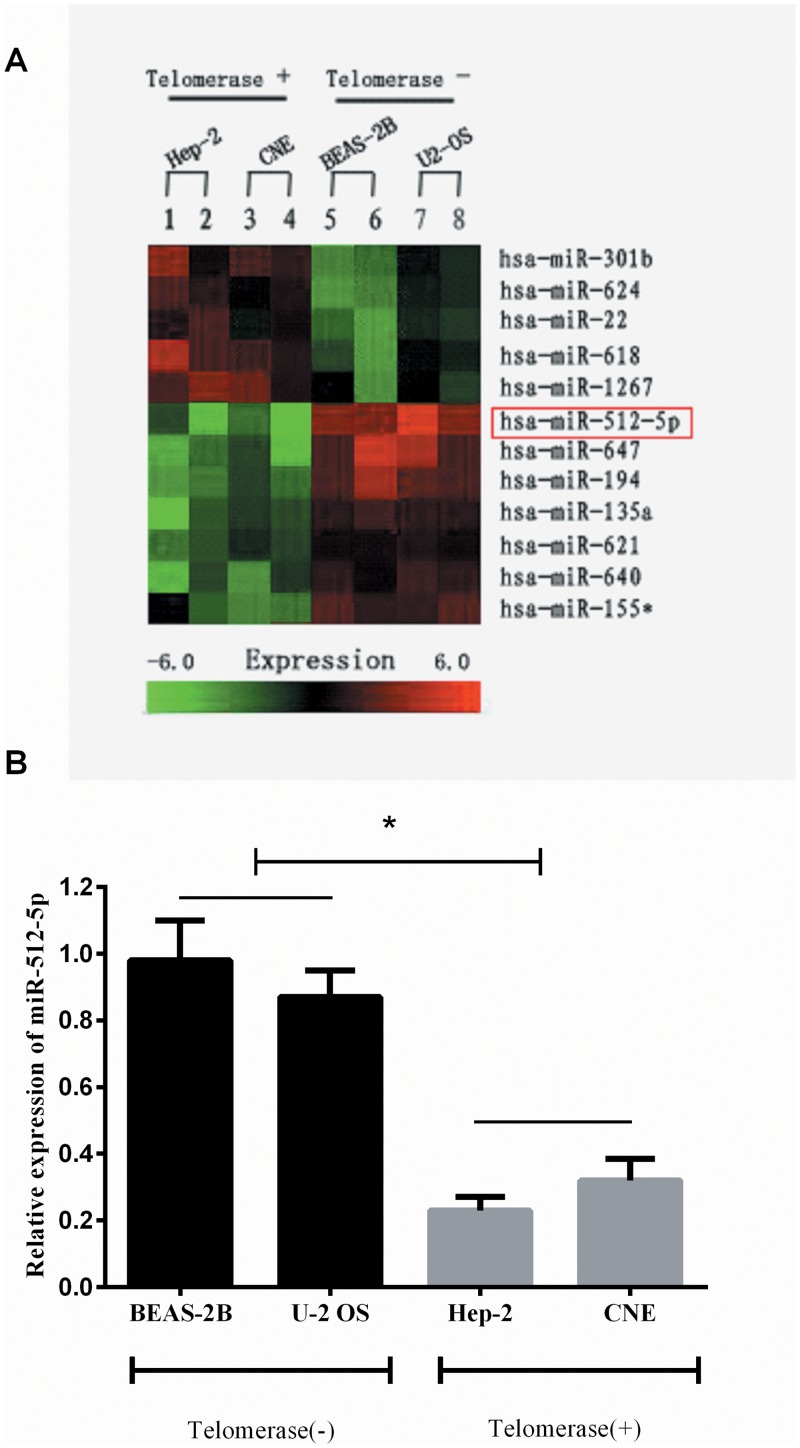
MiR-512-5p is down-regulated in in telomerase positive HNSCC cell lines. (A) MicroRNA array indicated that miR-512-5P was the most dramatically decreased among the miRs with significantly changed expression levels in telometase positive cell lines. (B) qRT-PCR analysis of the expression levels of miR-512-5p in various telomerase status cell lines. All data are shown as mean±SD. *P<0.05.

To determine the role of miRs in telomerase regulation, two telomerase positive cell lines (Hep-2, CNE) and two telomerase negative cell lines (BEAS-2B, U-2 OS) were assembled for microRNA profiling analysis. In telomerase positive cell lines7 miRs were down-regulated and 5 miRs were up-regulated compared to telomerase negative ones (Fig 1A and Table 1). Among the miRs with significantly changed expression levels, miR-512-5P was the most dramatically decreased by 0.13-fold (*p*<0.01). Consistent with the microRNA array results, QRT-PCR further validates that miR-512-5p levels were down-regulated in telomerase positive cell lines (Fig 1B). These findings suggested that miR-512-5p was reduced in telomerase positive HNSCC cell lines.

**Table 1:** Differentially expressed microRNA identified by microarray analysis.

miRNAs	Fold Change	
	Telomerase(+/-)	P value
**Overexpressed in Telomerase +**		
hsa-miR-301b	3.47	0.043
hsa-miR-624	2.83	0.011
hsa-miR-22	2.81	0.024
hsa-miR-618	2.36	0.032
hsa-miR-1267	1.65	0.037
**Underexpressed in Telomerase +**		
hsa-miR-512-5p	0.13	0.009
hsa-miR-647	0.31	0.018
hsa-miR-194	0.32	0.012
hsa-miR-135a	0.32	0.045
hsa-miR-621	0.35	0.029
hsa-miR-640	0.38	0.049
hsa-miR-155*	0.45	0.037

### MiR-512-5P suppresses HNSCC cell viability and induces apoptosis in vitro

**Fig 2:**
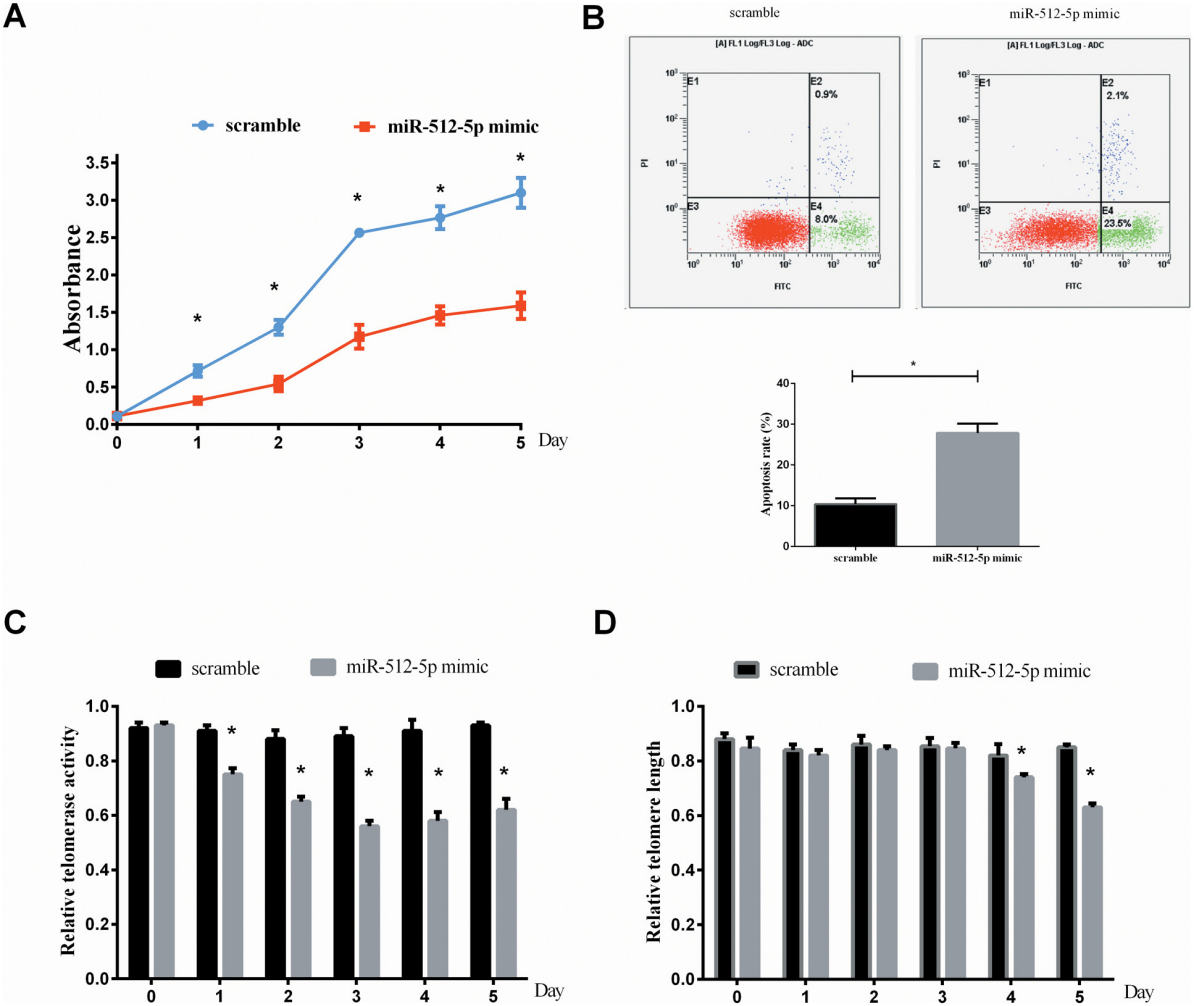
The effect of miR-512-5p on CNE cell growth, apoptosis and telomere maintenance. (A) MTT assay showed that overexpression of miR-512-5p in CNE cells resulted in inhibition of cell growth in vitro. (B) Forty-eight hours post-transfection,apoptosis was determined by flow cytometric detection of Annexin-V-FITC-positive/PI-negative cells. (C) One day post-transfection, a remarkable inhibition of telomerase activity was observed in cells transfected with miR-512-5P mimic in comparison with the cells transfected with scramble. (D) Four days post-transfection, miR-512-5p mimic groups showed a significant decrease in relative telomere length. All data are shown as mean±SD of triplicate experiments. *P<0.05.

In order to better characterize the impact of miR-512-5P on cancer cell proliferation, we introduced miR-512-5P mimics into the CNE cells. The transduction of miR-512-5P mimic showed a significant inhibition of cell proliferation in CNE cells, in comparison with those transduced with scramble as determined by MTT assay (Fig 2A). Furthermore, we observed that apoptosis rate weas significantly increased in miR-512-5P mimics-treated cells (Fig 2B). The results showed that up-regulation of miR-512-5P led to remarkable inhibition of cell proliferation and significantly increased apoptosis in HNSCC cells.

### MiR-512-5p suppresses telomerase activity and shortens telomere length in HNSCC cells

We next sought to explore whether miR-512-5P was capable of regulating telomerase activity in HNSCC cells, CNE cells were infected with miR-512-5P mimic and miR-512-5P-scramble, one day post-transfection, a remarkable inhibition of telomerase activity was observed in cells transfected with miR-512-5P mimic in comparison with the cells transfected with scramble, respectively (Fig 2C). These results indicated that miR-512-5p could suppress telomerase activity in telomerase positive HNSCC cells. We then address the potential effects of miR-512-5p on telomere length in telomerase positive HNSCC cells. Compared to the scramble groups, miR-512-5p mimic groups showed a significant decrease in relative telomere length 4 days post-transfection (Fig 2D). These results strongly suggested that miR-512-5p function in telomere maintenance involved telomerase and telomere length regulating.

### The hTERT mRNA is a target of miR-512-5p

**Fig 3:**
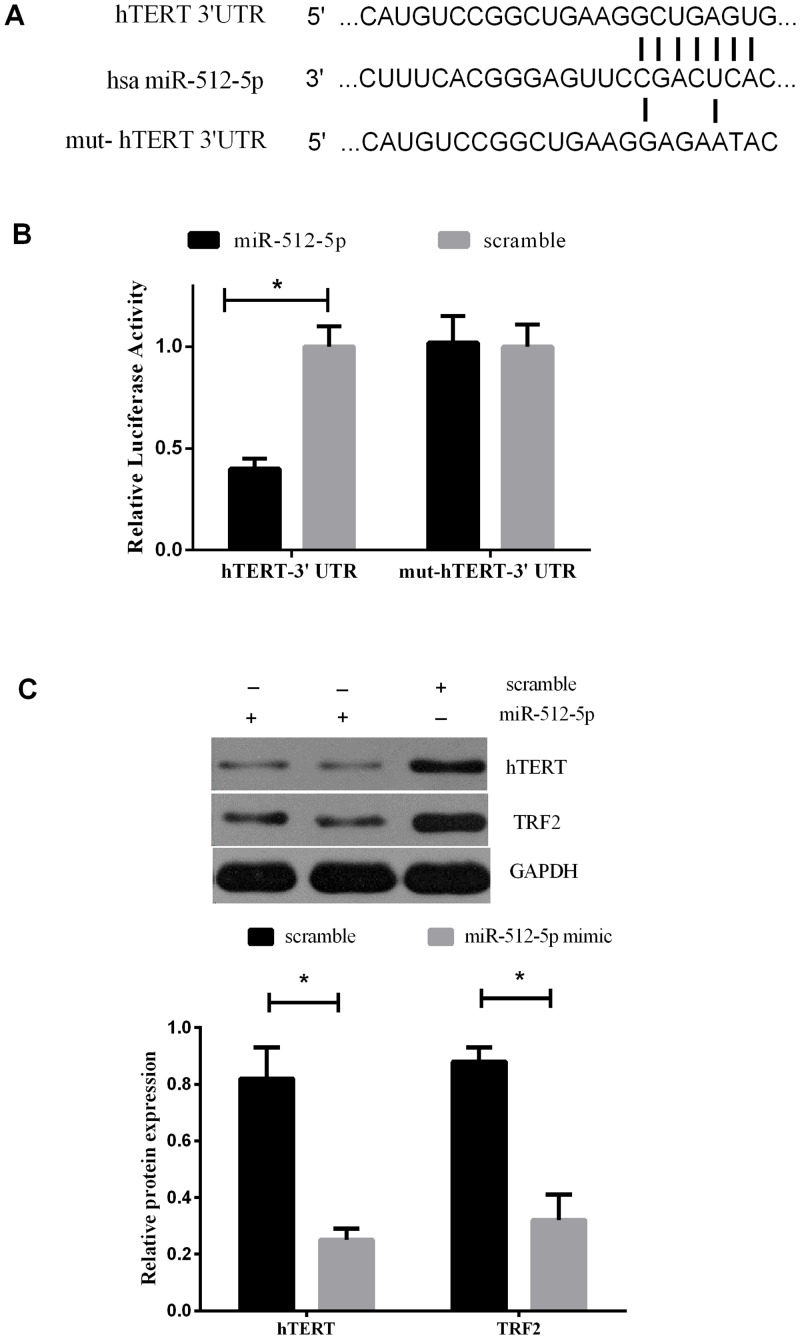
Validation of hTERT mRNA as a direct target of miR-512-5p in CNE cells. (A) Sequence of potential binding site of miR-512-5p in the 3’UTR of hTERT mRNA, mutations were introduced into the binding site for generation of mutated hTERT-3’UTR. (B) MiR-512-5p resulted in a significant decrease in luciferase expression in hTERT-3’UTR transfected cells compared with the scramble, while MiR-512-5p caused no statistical change in luciferase expression in mut-hTERT-3’UTR compared with the scramble(C) miR-512-5p mimic led to down-regulation of hTERT and TRF2 in CNE cells. All data are shown as mean±SD of triplicate experiments. *P<0.05.

We predicted potential direct targets of miR-512-5p by Targetscan Release 6.2., PicTar5 and miRanda 3.0 programs (S1 Table). The hTERT gene was predicted to have at least one potential binding site at their 3’-UTRs for miR-512-5p (Fig 3A). To validate whether miR-512-5p directly repress identified mRNA targets through 3’UTR interactions, we cloned a sequence with the predicted target sites of hTERT or a mutated sequence to downstream of the pMIR luciferase reporter gene. The above plasmids and miR-512-5p mimic or miR-512-5p-scramble were transiently transfected into 293T cells and a dual luciferase reporter assay system was used to detect luciferase expression 48 h after tranfection. The results indicated that overexpression of miR-512-5p resulted in a significant decrease in luciferase expression in hTERT-3’UTR transfected cells, compared with the scramble. However, transfection of miR-512-5p in mut-hTERT-3’UTR transfected cells did not display significant reduction of luciferase levels (Fig 3B). We further investigated the effect of miR-512-5p on the expression of hTERT and TRF2 by Western blot. Obviously, the result showed that miR-512-5p mimic led to down-regulation of hTERT and TRF2 in CNE cells (Fig 3C). These data demonstrated that miR-512-5p regulated hTERT expression at the post-transcriptional level and influenced the telomere-binding proteins.

### MiR-512-5p suppresses tumor growth and hTERT expression in vivo

**Fig 4:**
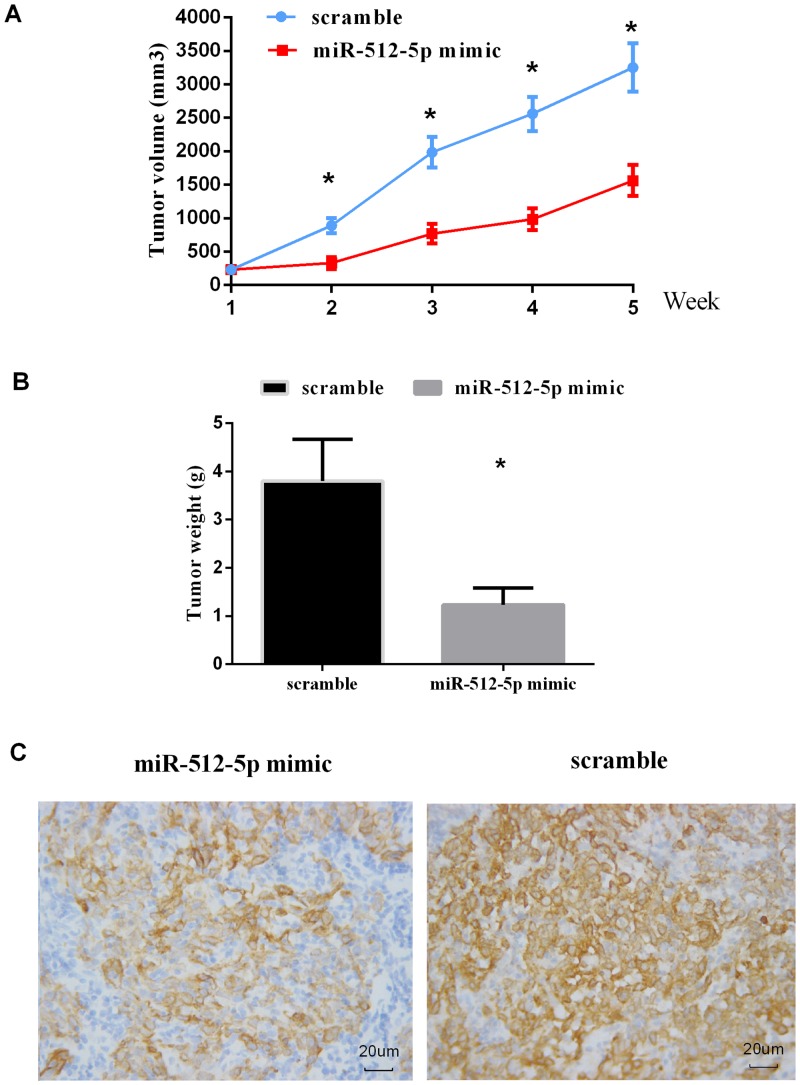
In vivo effect of miR-512-5p on CNE cell growth in nude mice. Tumor size was measured weekly using method mentioned above. (A) Growth curves for CNE/miR-512-5p mimic (n = 10) or CNE/scramble (n = 10) cells by in vivo proliferation assay. (B) Tumors were weighed after animals were killed at 5 weeks after injection. The weight of tumors was significantly decreased in miR-512-5p group compared to scramble group (*p* = 0.009). (C) Representative photomicrographs of immunohistochemical staining of TERT on xenograft tumor sections obtained from mice in miR-512-5p and scramble groups by immunohistochemistry at 400× magnifications. All data are shown as mean±SD. *P<0.05.

To investigate the in vivo effect of miR-512-5p, equal numbers of CNE cells treated with miR-512-5p or the scramble were subcutaneously injected into nude mice and immunohistochemistry assays for hTERT were performed. Data of tumor volume (Fig 4A) showed that CNE cells treated with scramble led to larger tumors more rapidly than miR-512-5p-transfected cells in nude mice(P<0.01). Similarly, compared to the scramble group (3.81±0.87 g), mice injected with miR-512-5p mimic transfected cells (1.23±0.35 g) showed a significant decrease in tumor weight (Fig 4B). Furthermore, the expression of hTERT protein was dramatically down-regulated in the miR-512-5p treatment group compared with the scramble group (Fig 4C). These results demonstrated that miR-512-5p suppresses tumor growth and hTERT protein expression in vivo.

## Discussion

Telomerase is a RNA-dependent DNA polymerase that synthesizes telomeric repeats at the 3’-end of the leading DNA strand [14, 15]. Telomerase activity, which is essential for the maintenance of tumor cell immortality, is required for the survival of the majority of tumor cells probably critical for sustained tumor proliferation[16]. Therefore, telomerase seems to be a novel target for cancer therapeutics[6]. Telomerase is a ribonucleoprotein consisting of the RNA component, hTR, which provides the template for DNA synthesis, and the catalytic subunit, human telomerase reverse transcriptase (hTERT), which provides the catalysis of telomerase and regarded as the determinant component to maintain the telomerase activity, so,suggesting that hTERT is the key component for the control of telomerase activity[3]. In our previous studies, we have demonstrated that hTERT is mostly transcriptionally regulated [17–19]. However, recent studies have indicated that hTERT can also be regulated by post-transcriptional mechanisms [20, 21]. In addition, many other accessory proteins, such as nuclear factor-κB[22], can modulate telomerase activity by interaction with hTERT. Therefore, post-transcriptional factors that are involved in the regulation of telomerase have generated considerable interest, and miR are now considered to play a key role in the post-transcriptional mechanism[9].

MicroRNAs are a class of non-coding, small RNAs that regulate gene expression by promoting decay or suppressing translation of their target messenger RNAs at the post-transcriptional level[10]. MiRs play crucial roles in the initiation and promotion of cancer by regulating the expression of multiple oncogenes or tumor suppressors[23]. The results of this study indicated telomerase-positive cells revealed significantly lower levels of miR-512-5p expression compared with telomerase-negative cells. In addition, overexpression of miR-512-5p in HNSCC cells could suppress cell proliferation and increase apoptosis in vitro and suppress tumor growth in vivo. Meanwhile, previous study reported activation of miR-512-5p by epigenetic treatment induces suppression of Mcl-1, resulting in apoptosis of gastric cancer cells [24]. Our findings demonstrate that miR-512-5p has properties consistent with tumor suppressor function in HNSCC. It is thus of importance to recognize miR-512-5p as a potential useful tumor suppressor gene.

We next explored the possible targets of miR-512-5p in HNSCC cells through different computational programs. The prediction of potential targets revealed hTERT as a candidate target of miR-512-5p, which attracted our attention. Next Luciferase reporter assay was performed to demonstrate hTERT as a direct target of miR-512-5p in HNSCC cells. As one of the most important structure of telomerase, hTERT have been relevant with the progression of histological dedifferentiation, tumor stage, and malignant behavior such as invasive and metastatic phenotypes in a number of previous reports [25–27]. Recent evidence shows that hTERT may promote tumor growth by maintaining telomerase activity, telomere-binding proteins and telomere length [5, 6, 8]. Furthermore, additional studies indicate that hTERT modulates the expression of apoptotic genes and enhances cell proliferation[8]. In our study, the ectopic expression of miR-512-5p inhibited the HNSCC cell growth by down-regulation telomerase activity, telomere length and telomere-binding proteins, and the delivery of miR-512-5p into transplanted tumors in nude mice significantly reduced tumor growth. These research data suggest that the delivery of miR-512-5p could represent a novel therapeutic strategy in HNSCC therapy by targeting hTERT. With respect to the relationship of miR-512-5p and hTERT, in-depth studies might give us more information for better understanding the roles of miR-512-5p on telomere maintenance and tumorigenesis in HNSCC, which is needed to be further investigated.

In conclusion, we here report expression patterns of altered miRs in HNSCC and the potential role of miR-512-5p in telomerase-positive HNSCC cells. Overexpression of MiR-512-5p significantly suppress HNSCC cell proliferation by targeting hTERT in vivo and in vitro, our current results strongly suggest that miR-512-5p might serve as a potentially therapeutic agent for miRNA-based HNSCC therapy.

## Supporting Information

S1 Table The list of the predicted target genes of miR-512-5P from the Targetscan (Release 6.2), PicTar5 and miRanda 3.0 programs. (XLSX)
